# Comprehensive Insights Into Basal Cell Carcinoma: Causes, Presentation, Prevention, and Modern Therapeutic Approaches

**DOI:** 10.1002/cam4.71448

**Published:** 2025-12-19

**Authors:** Sophia Levit, Joshua Shoykhet, Eyal Levit

**Affiliations:** ^1^ Georgetown University Washington District of Columbia USA; ^2^ CUNY Hunter College, Bachelor of Arts New York New York USA; ^3^ Columbia University College of Physicians & Surgeons New York New York USA

**Keywords:** dermatoscopy, nicotinamide prophylaxis, nonmelanoma skin cancer, skin of color, sonic hedgehog inhibitors

## Abstract

**Background:**

Basal cell carcinoma (BCC) is the most common skin cancer worldwide, accounting for approximately 80% of all skin cancers. Although more prevalent among Caucasians, BCC affects individuals of all racial and ethnic groups and may present atypically in skin of color, contributing to delayed diagnosis.

**Aims:**

This review aims to examine the causes, clinical presentations, prevention strategies, and treatment options for BCC, with an emphasis on recent advancements and ongoing challenges in management.

**Materials and Methods:**

This review integrates current peer‐reviewed literature with clinical insights derived from dermatologic practice, including the evaluation of representative, deidentified patient cases. Observations from clinical experience were incorporated to contextualize diagnostic approaches, management strategies, and treatment outcomes across diverse patient populations. All patient information was anonymized, and no identifiable data were included.

**Results:**

Ultraviolet (UV) radiation remains the leading cause of BCC in fair‐skinned populations, whereas genetic predispositions and environmental factors play a larger role in darker‐skinned individuals. Diagnostic advances, including dermoscopy and reflectance confocal microscopy, have improved detection, particularly for pigmented lesions that may mimic benign conditions. Preventative strategies include broad‐spectrum physical sunscreens, protective clothing, lifestyle modifications, and supplementation such as nicotinamide, which has demonstrated potential in reducing UV‐induced skin damage. Treatment options range from Mohs micrographic surgery, considered the gold standard for high‐risk and cosmetically sensitive areas, to simple excision, electrodessication and curettage, radiation therapy, and advanced systemic therapies including sonic hedgehog and PD‐1 inhibitors for locally advanced or metastatic disease.

**Discussion:**

Emerging diagnostic tools and expanding therapeutic modalities have enhanced the ability to tailor BCC management to individual patient risk profiles and tumor characteristics. Non‐surgical and combination approaches continue to evolve, offering effective alternatives for select cases.

**Conclusion:**

By integrating prevention, early diagnosis, and personalized treatment strategies, modern approaches to BCC management are improving patient outcomes and addressing the complexities of care across diverse populations, underscoring the importance of multidisciplinary and individualized management.

## Graphical abstract

This article provides a comprehensive overview of basal cell carcinoma (BCC), focusing on innovative non‐surgical prevention and treatment protocols. It introduces a novel, practitioner‐friendly algorithm for BCC management, offering clear guidance for selecting treatment based on patient profiles and tumor characteristics. The review emphasizes prevention strategies, including supplementation to reduce UV‐related damage, aiming to enhance patient outcomes and support early intervention in skin cancer care.

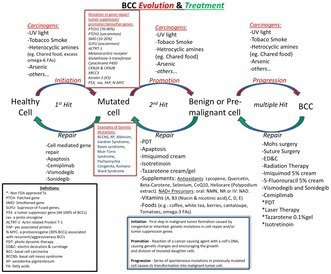

## 1 Introduction

The first recorded case of skin cancer was in a 24‐year‐old mummy found in Peru [1]. Today, as was likely in past human history, skin cancer remains the most common type of cancer worldwide, with 3.5 million new cases diagnosed annually in the U.S. alone [1]. Of those, approximately 80% are basal cell carcinomas (BCC), making it the most common type of skin cancer across all racial and ethnic groups [1].

While the incidence of skin cancer, including BCC, is significantly higher among Caucasians, it remains an important health concern in individuals of all skin tones. BCC still constitutes a significant portion of skin cancers in people of color, often presenting differently and leading to delayed diagnoses. According to the American Academy of Dermatology (AAD), BCC incidence rates vary across racial and ethnic groups:

- *Caucasians:* 75%–80% of skin cancer cases are BCC [2].
- *Hispanics:* BCC represents about 5% of all cancers [3].
- *Asians:* 2%–4% of cancers [3].
- *African Americans:* 1%–2% of cancers, though cases tend to present later and more aggressively [3].

BCC is more common in sun‐exposed areas such as the head and neck but can develop in areas with minimal sun exposure due to genetic predispositions, environmental exposures, and immunosuppression [2].

## 2 Causes of BCC

The causes of BCC are similar across all skin types, though risk factors may vary. Ultraviolet (UV) radiation is the leading cause in lighter‐skinned populations, while genetic predispositions and environmental exposures play a larger role in darker‐skinned populations [2]. Risk factors include (see Table 1).

- UV radiation (especially in fair‐skinned individuals)
- Scarring or trauma.
- Genetic disorders like xeroderma pigmentosum and nevoid BCC syndrome (See Table 1, for a full list of genetic disorders associated with BCC)
- Immunosuppression
- Carcinogens: most commonly Arsenic.

**TABLE 1:** Pathogenesis and treatment options for BCC evolution and treatment.

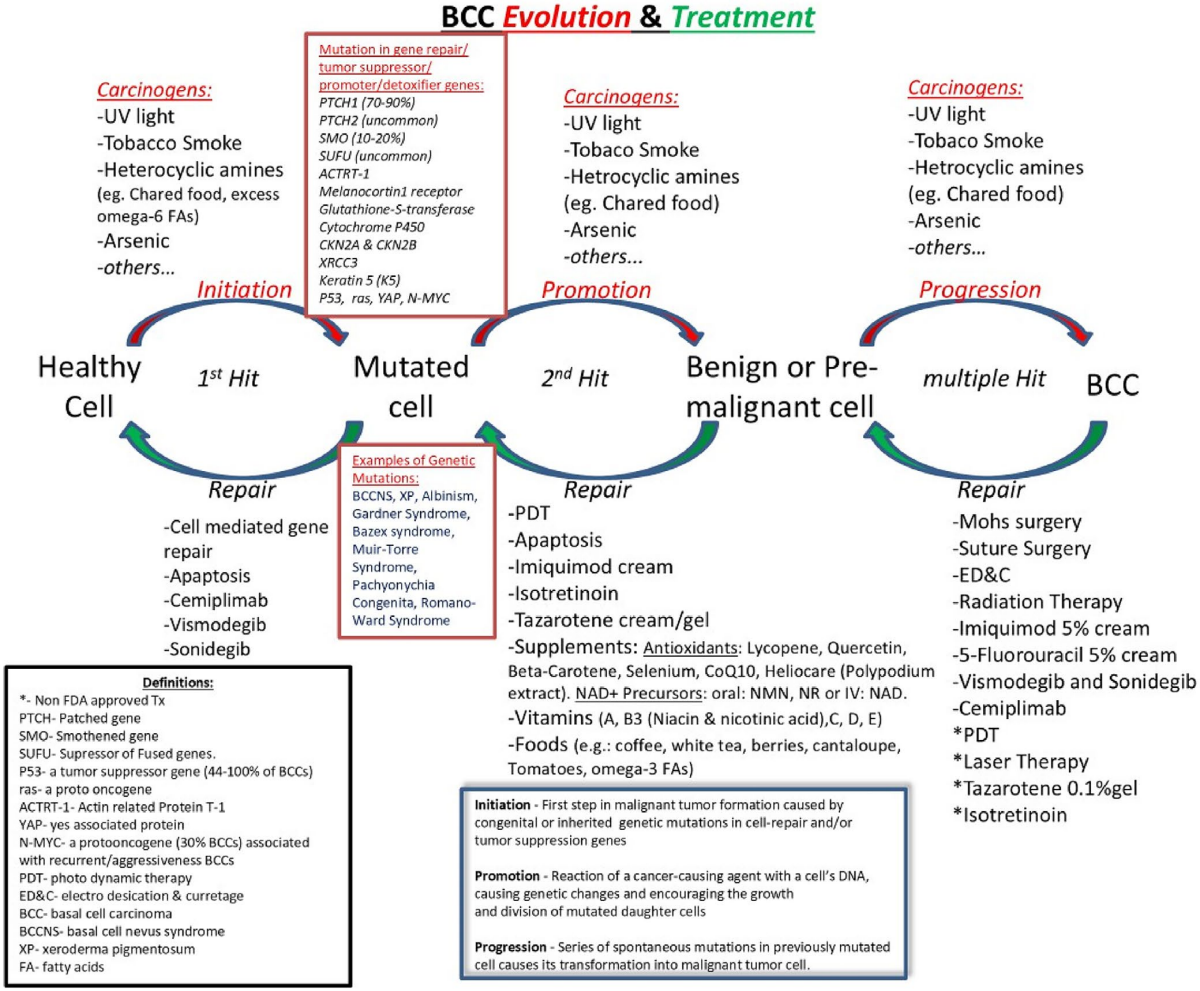

### 2.1 Clinical Presentation

**FIGURE 1:**
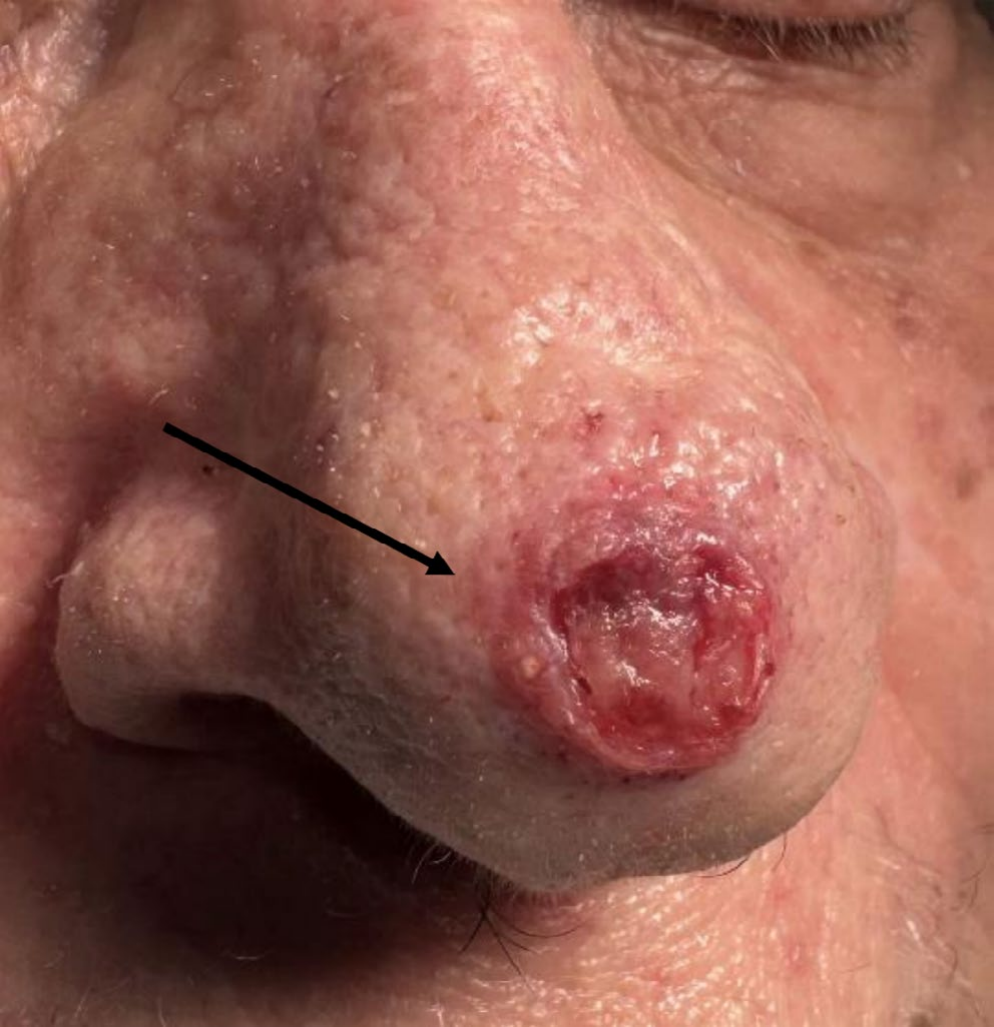
(See black arrow) Ulcerated Nodular basal cell carcinoma on the nasal tip of a caucasian female.

BCC can present differently depending on skin tone:

- In Caucasians, it typically appears as a pearly or telangiectatic nodule, often with ulceration [2] (See Figure 1).
- In darker‐skinned individuals, it often appears as pigmented papules or plaques, which can be misdiagnosed as seborrheic keratosis, benign moles, or even malignant melanoma. This leads to delayed diagnosis and more advanced disease at presentation [1].

### 2.2 Dermoscopy of BCC

- Diagnosis of BCCs can be aided with noninvasive technologies such as dermoscopy and reflectance confocal microscopy (RCM). The key signs of BCC seen on dermoscopy include arborizing telangiectasia, which is defined as thin or thick branched blood vessels resembling tree branches, and the absence of a pigment network, which helps differentiate basal cell cancer from a typical nevus. RCM's large field of view allows for the evaluation of larger areas than routine histopathological analyses, which can help create a more effective treatment plan [4]. RCM's drawback is that its imaging resolution decreases once a depth of 100–150 μm is reached, making it more difficult to detect tumor invasion. Dermascopy takes significantly less time than RCM, therefore it can be used for preliminary evaluation of skin lesions and to determine if RCM is needed. In darker skin tones (phototypes II‐IV) or pigmented lesions, RCM uses melanin's natural contrast to clearly identify basal keratinocytes and melanocytes [4]. Thus, RCM is an effective tool to diagnose skin cancers, especially in people of color.

### 2.3 Prevention

Prevention of BCC largely focuses on minimizing exposure to risk factors, particularly UV radiation from the sun and tanning beds.

1. *Sunblock/Sunscreen:* A Broad‐spectrum sunblock with an SPF of 30 or higher is recommended. Ideally, a physical sunblock that can protect the skin in the UVB, UVA, and blue light range should be used. Zinc Oxide and/or Titanium dioxide for UVB and UVA protection, as well as iron oxide, a mineral helping to reduce radiation from UVA and blue light, are key ingredients to look for in a good sunblock. The particle size matters. While micronized particles improve esthetics, smaller particles, such as nanoparticles, could potentially enter the bloodstream and should be avoided. A proper amount of sunblock must be applied, and the authors thus prefer a tinted sunblock in cream form to visualize where the sunblock was applied. Using a spray‐type sunblock, although popular and convenient, does not provide sufficient coverage and may be more hazardous if it penetrates the airways. The sunblock should be applied before the individual leaves the home. One must remember that UVA radiation penetrates through glass, and thus sunblock should be used even when protected by clear glass or in the shade (due to UV reflection from other objects). Sunblock must be applied generously and frequently, especially when spending extended time outdoors.
2. *Protective Clothing:* Long‐sleeved shirts, wide‐brimmed hats, and sunglasses can help protect the skin from UV rays. A detergent additive called SunGuard can be added to the laundry, providing regular clothes with UPF (Ultraviolet Protection Factor) to help increase the fabric's ability to block UV rays, effectively giving everyday clothing a degree of sun protection comparable to an SPF of 30 that is sustained for about twenty wash cycles.
3. *Seeking Shade:* Direct sunlight during peak UV hours, typically between 10 a.m. and 4 p.m. is most harmful.
4. *Avoiding Tanning Beds:* Tanning lamps, as well as UV machines used for nail gel manicures, are sources of UV radiation that can increase the risk of skin cancer both through mutations in the DNA and through suppression of the CD‐8 T‐cell immune surveillance [5]
5. *Regular Skin Exams:* Regular self‐exams are helpful in identifying any new or changing lesions that might suggest skin cancer. A yearly visit to a dermatologist for regular skin checks should be recommended to all patients.
6. *Supplements* (see Table 1): Although controversial, supplements have been recommended and have shown some efficacy in reducing UV‐induced damage and are thus believed to help reduce the risk of non‐melanoma skin cancers. A 1‐year longitudinal study revealed the efficacy of Nicotinamide (500 mg twice daily), a vitamin B3 derivative, in reducing the numbers of precancers and non‐melanoma skin cancers in high‐risk individuals by about 23% [6]. Other agents are believed to function by reducing oxidative stress caused by UV radiation, they include, among many, Polypodium extract (marketed as Heliocare) and beta carotene. A word of caution: while this may be of help in the skin, a study using Carotene and retinol CARET (Carotene and Retinol Efficacy Trial) found that beta‐carotene supplements did not prevent but increased the risk of lung cancer and heart disease in high‐risk groups like smokers and asbestos‐exposed workers [7]. Thus, caution must be exercised when recommending high‐dose beta‐carotene supplementation, particularly among smokers

## 3 Treatment Options

See Tables 2, 3, 4 for the algorithm and history of BCC treatment.

**TABLE 2:** Stratification algorithm for low versus high‐risk BCC.

Risk level	Location	Tumor diameter	Histological variant	Immunosuppression	Recurrence factor
High	Area H (central face, eyelids, eyebrows, periorbital skin, nose, lips, chin, mandible, preauricular and postauricular skin/sulci, temple, ear, genitalia, hands, and feet), area of prior radiation Area M (cheeks, forehead, scalp, neck, and pretibial)	Area H *or* prior radiation Any size. Area M ≥ 1 cm	– Morpheaform – basosquamous – sclerosing – mixed infiltrative – micronodular features in any portion of the tumor	Yes [History of immunosuppression (e.g., organ transplant, HIV/AIDS)]	Yes (Tumor recurrence following prior therapy)
Low	Area L (trunk and extremities)	< 2 cm (*If* ≥ *2 cm consider as high risk*)	– keratotic – infundibulocystic‐fibroepithelioma of Pinkus	No (No significant immunosuppression)	No (primary tumor not treated before.)

*Note:* Once the tumor is defined as high or low risk, one can follow the flow chart below for further guidance with more detail in the therapy list mentioned above under Treatment options.

**TABLE 3:** Management algorithm for BCC.

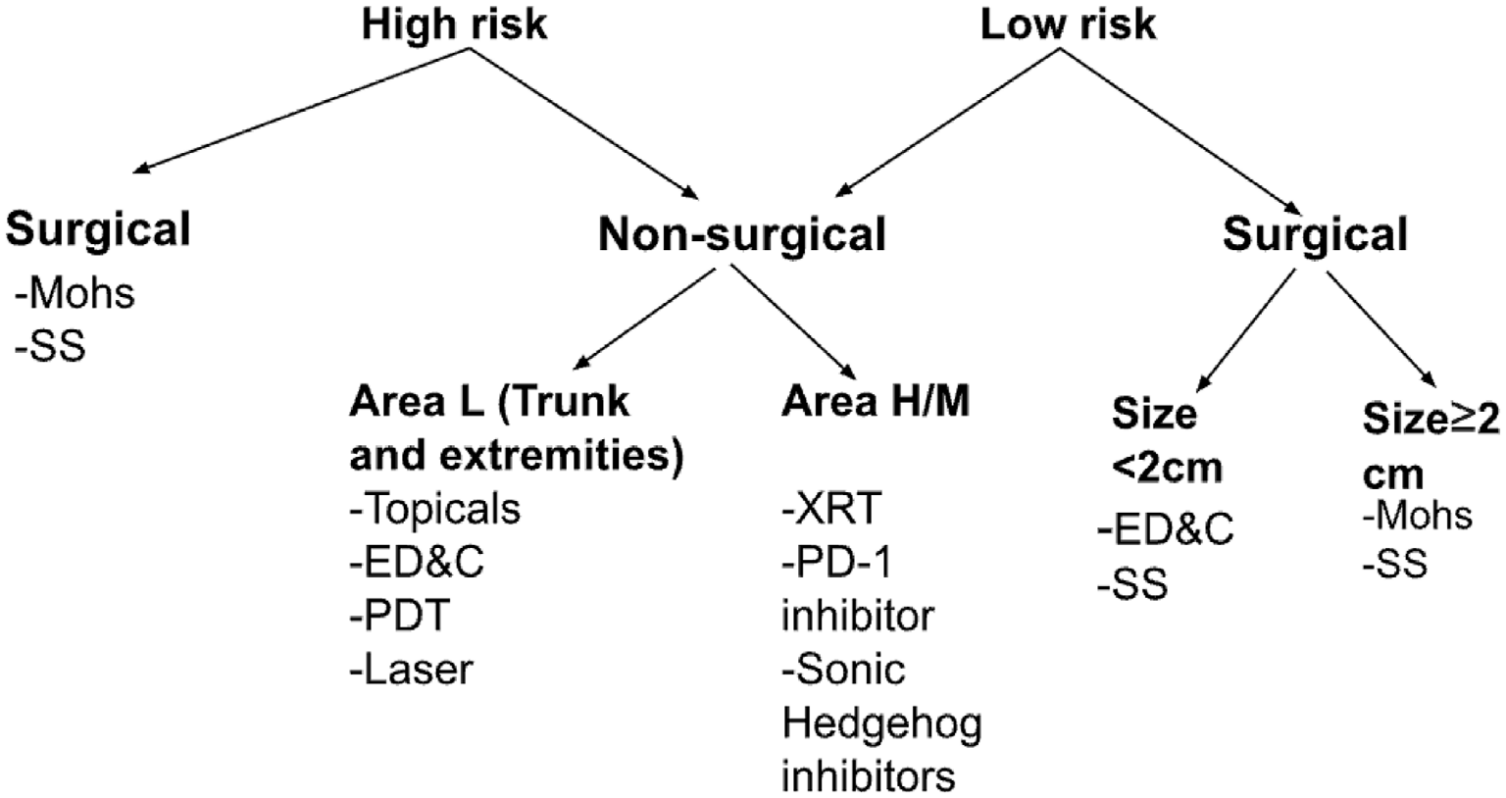

Abbreviations: Area H/M, Central face, eyelids, eyebrows, periorbital skin, nose, lips, chin, mandible, preauricular and postauricular skin/sulci, temple, ear, genitalia, hands, and feet, cheeks, forehead, scalp, neck and pretibial regions; ED&C, electrodessication & curettage; Nonsurgical candidate is defined as: High risk for complications from surgery (bleeding, anesthesia, wound care) or refuses surgery; PDT, photodynamic therapy; SS, suture surgery AKA excisional surgery.

**TABLE 4:** History of BCC therapy.

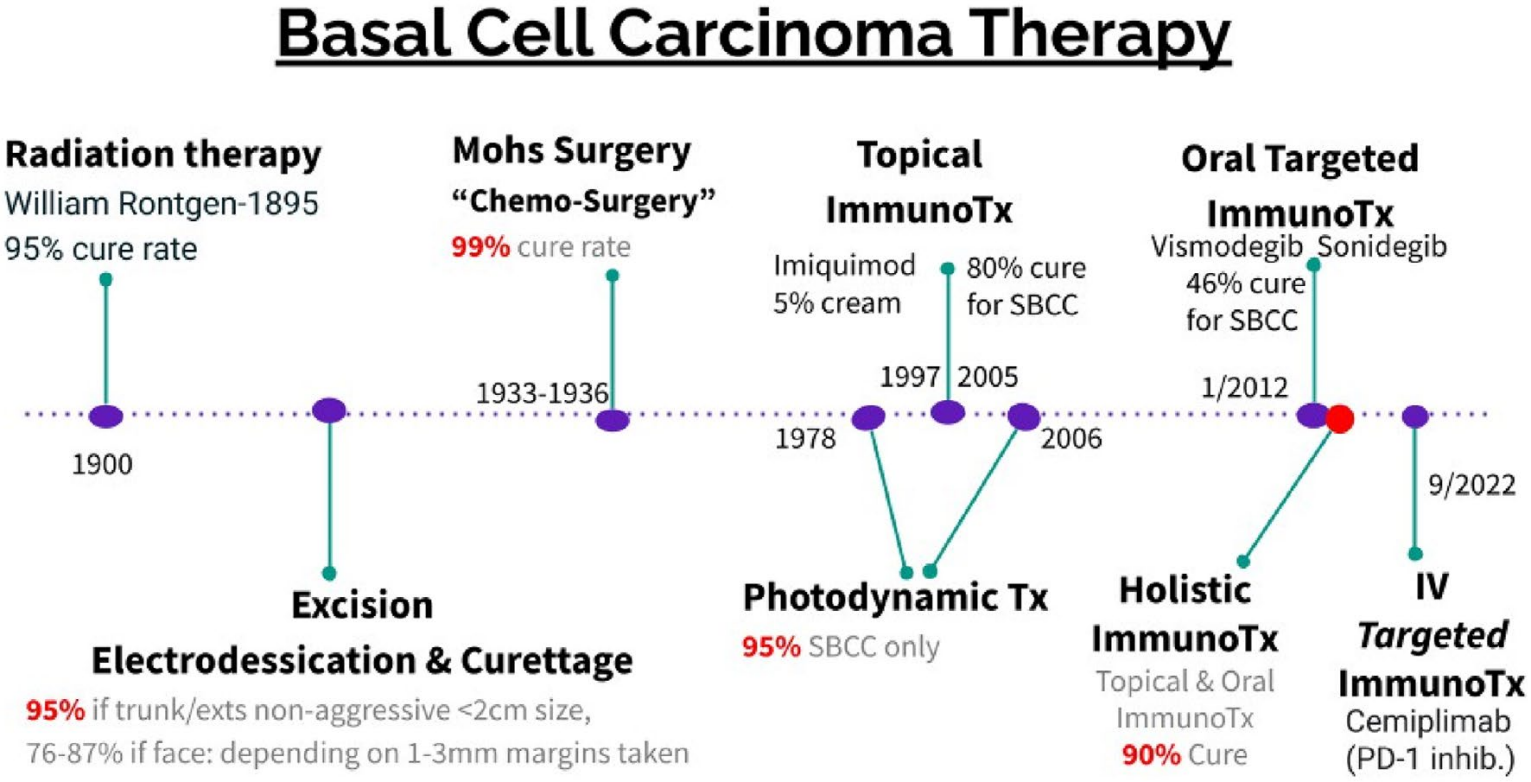

Abbreviations: Holistic ImmunoTx, refers to the combination of oral targeted immunotherapy with topical Imiquimod therapy as described in the article above; ImmunoTx, Immunotherapy; IV, Intravenous; PD‐1 inhibitor, Program Cell Death‐1 inhibitor; SBCC, Superficial BCC; Tx, Treatment.

* This table is taken from a presentation by Levit et al. [8].

### 3.1 Mohs Surgery

**FIGURE 2:**
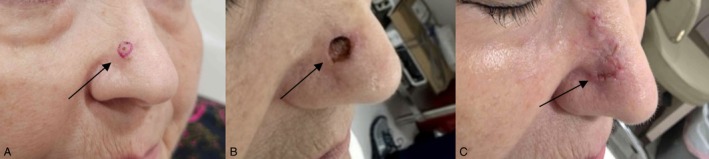
(See black arrow on all images) (A) Mixed Nodular and Infiltrative BCC, marked with a red sharpie pen indicating presumed cancer size, on the right nasal sidewall of a caucasian female before biopsy. (B) Mohs defect revealing true cancer size. (C) Scars appearance at suture removal 9 days after repair with successful conservation of the nasal cosmetic and functional units.

Mohs surgery is the gold standard for treating BCC, particularly for lesions on the face, neck, hands, feet, and genitals. Its microscopic precision ensures the complete removal of cancer cells while sparing healthy tissue. Mohs surgery boasts a 99% cure rate (See Figure 2) [1].

### 3.2 Suture Surgery (Simple Excision)

This is a standard surgical procedure where the lesion is excised using clinical judgment. The resulting defect is closed with sutures, achieving optimal healing and cosmesis. Suture surgery has a 98% efficacy rate in treating BCC^2^ but that depends on the margins removed and depth with the current recommendation being 4 mm margins to the subcutaneous fat [9]. This cure rate is reduced, the larger the original size of the BCC is, and thus for BCCs greater than two centimeters even on the trunk or for recurrent BCCs Mohs surgery is the preferred surgical treatment option for surgical candidates (See Tables 2, 3, 4).

### 3.3 Electrodessication and Curettage (ED&C)

**FIGURE 3:**
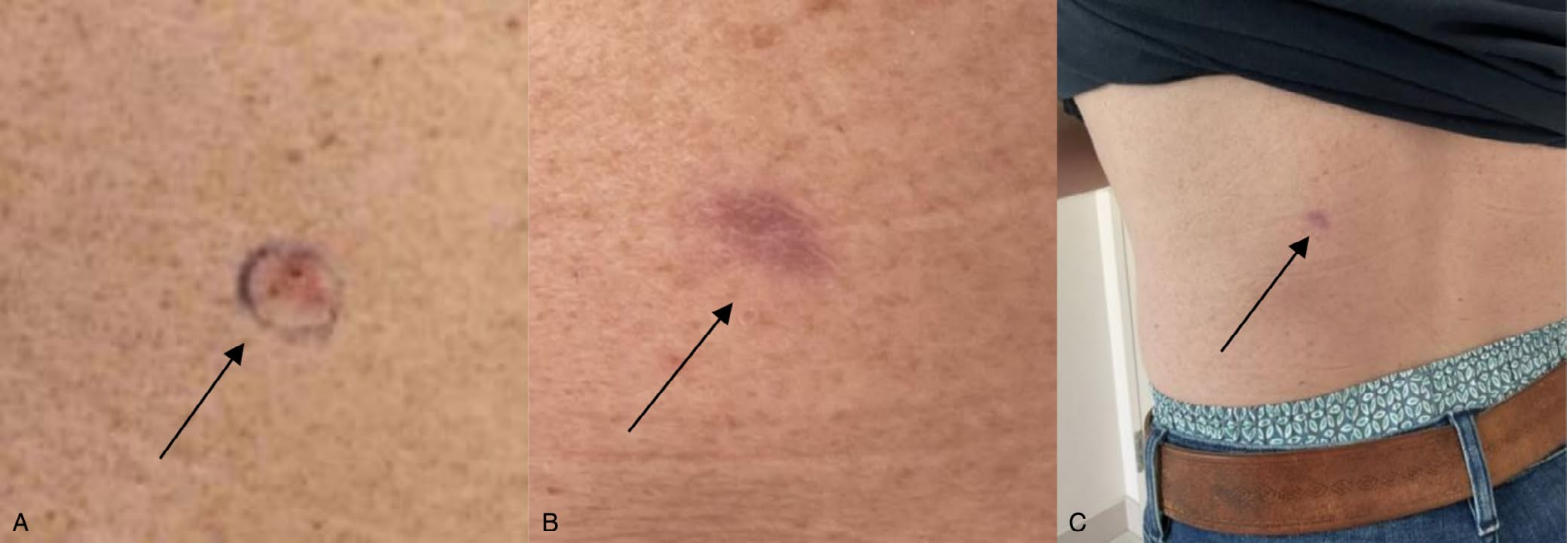
(See black arrow for each picture A‐C). (A) A superficial basal cell carcinoma on the left lower back of a caucasian male before ED&C × 3 cycles. (B) The area four months post ED&C followed by 8 weeks daily application of Imiquimod 5% cream to the procedure site in order to reduce scarring. (C) A full profile of the left lower back after completing the treatment.

ED&C involves scraping away the cancerous tissue using a curette followed by electrocautery to stop bleeding and destroy residual cancer cells. It has a 93%–98% success rate, depending on the treating physician and the area treated with higher cure rates on trunk and extremities (Low‐risk, non‐facial areas) [1]. The drawback is that it takes a few months to heal and leaves a depigmented, often depressed round scar in the treatment site and thus should be reserved for non‐cosmetically sensitive areas [9]. Higher cure rates have been reported by using a smaller‐sized curet with more aggressive scraping and cautery and by increasing the times each curettage and electrocautery cycle is performed during the same visit from 3 cycles to five (See Figure 3).

### 3.4 Radiation Therapy (XRT)

**FIGURE 4:**
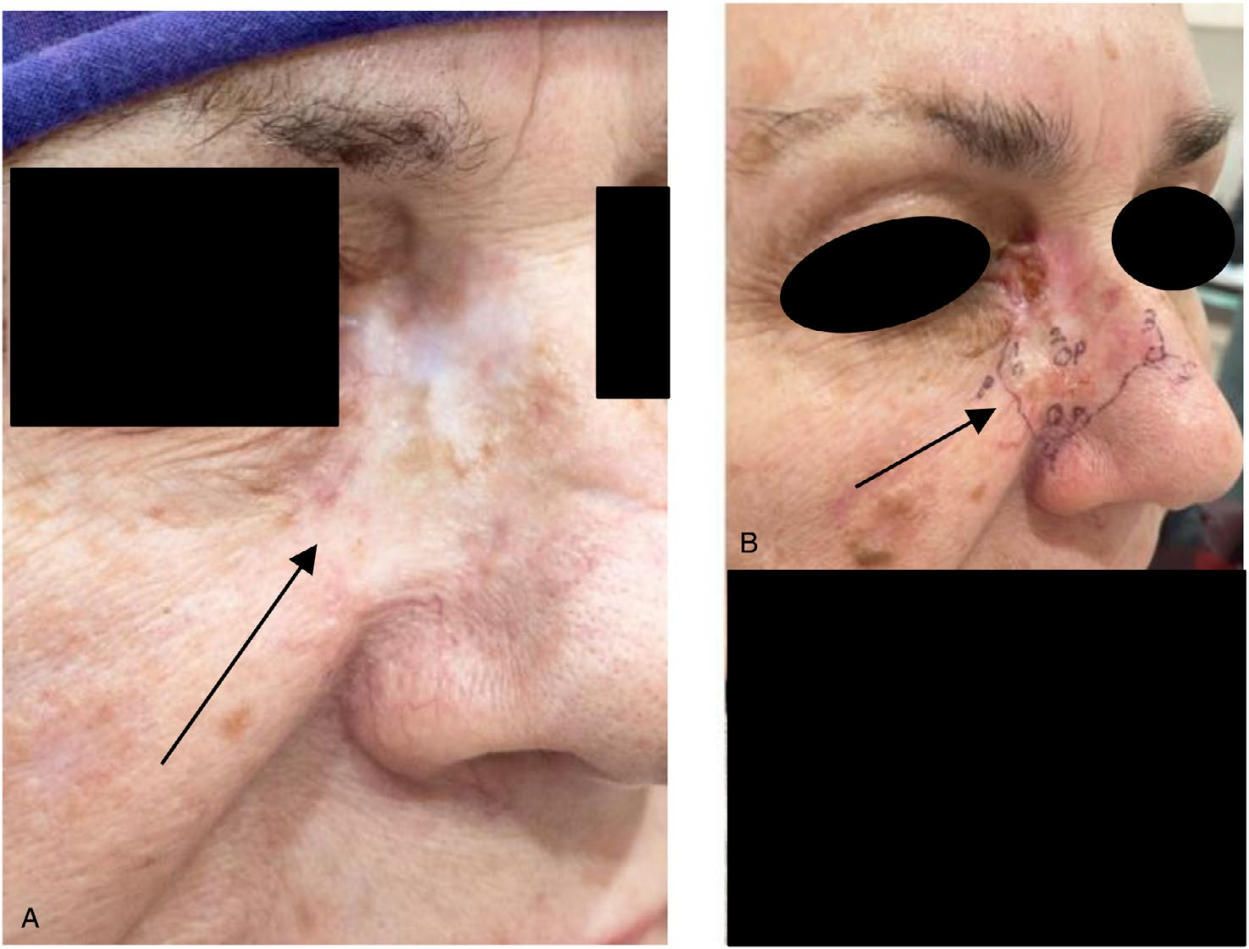
(A) scarred skin after radiation therapy for BCC (black arrow). (B) Recurrent morpheaform BCC with areas of SCC (squamous cell carcinoma) on the right nose ~10 years after radiation treatment (see arrow and black skin marker surrounding the tumor's extent). The problem is not only to detect recurrence of the cancer that appears like a scar thus difficult to differentiate from the radiation scar. But if it recurs reconstructing this poorly perfused scarred down skin can be a surgical nightmare.

Radiation therapy, including both electron beam and photon (X‐ray) radiation, is an effective treatment option for patients who cannot undergo surgery or prefer not to. It boasts a high cure rate, often around 95%, particularly for BCC [2]. However, this treatment carries risks, including the potential development of secondary skin cancers 20 years post‐treatment. Additionally, scarring from radiation therapy can complicate the identification and management of any future recurrences, making them more difficult to treat (See Figure 4).

### 3.5 Sonic Hedgehog Pathway Inhibitors

Vismodegib (Erivedge) and Sonidegib (Odomzo) are oral medications approved for locally advanced or metastatic BCCs that cannot be treated surgically. These inhibitors target the hedgehog signaling pathway, which is abnormally activated in most BCCs [10].

**FIGURE 5:**
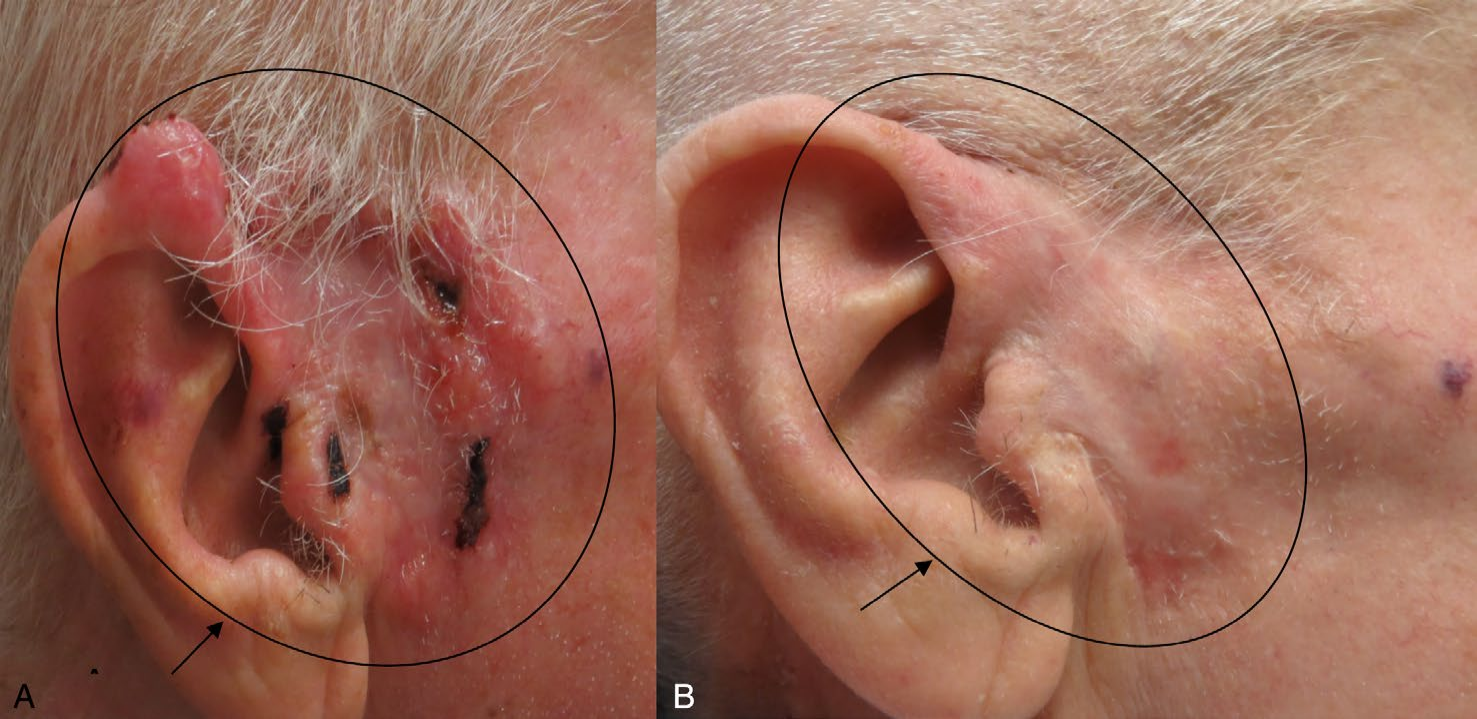
(A) Nodular Basal Cell Carcinoma, Pre‐treatment (see arrow pointing to the ellipse of the BCC plaque). (B) 6 months Post‐treatment cessation. Clearance was confirmed by multiple scouting punch biopsies, 6 months after completing a combination of Vismodegib 1×/day for 4 months and Imiquimod 5% cream daily for 6 weeks (See black arrow pointing to the ellipse of the resolved BCC).

- *Vismodegib*: Typically taken once daily, vismodegib has a half‐life of 4 days [11]. The most common side effects include muscle cramps, taste disturbances, and hair thinning [11]. This medication can be taken with or without food. A study showed that after a 6 month treatment period, 31% of patients with complete remission of the disease reported recurrence later on [10]. A new treatment method developed by the author E.L. and used since 2012 combines Erivedge and Aldara, resulting in a 90% cure rate (See Figure 5) [8].
- *Sonidegib*: Taken once daily, sonidegib has a longer half‐life of 28 days and should be taken on an empty stomach to avoid decreasing its absorption [11]. Side effects are similar to vismodegib.

### 3.6 Dose Adjustments and Practical Considerations

In cases where patients are taking other medications that interact with the cytochrome P450 (CYP3A4) enzyme pathway—such as certain antifungals (e.g., ketoconazole) or antibiotics (e.g., clarithromycin)—the metabolism of these hedgehog inhibitors may be slowed, increasing the risk of side effects [11]. This can lead to more severe and prolonged side effects such as muscle cramps, fatigue, and taste disturbances, which typically occur between the second and fourth day after taking the medication, leading to a discontinuation rate of 20%–30% [8] Although not reported, the author E.L. has noticed a few cases of sudden loss of consciousness and falls otherwise unexplained except by the medication.

To manage this, dose adjustments are often necessary. In the author's E.L. practice, he sometimes reduces the dose to once a week or even prescribes half or one‐third of a pill per week to minimize side effects while still achieving therapeutic benefits [8]. This strategy helps patients tolerate the medication better, reducing the generally high discontinuation rate seen with the current daily FDA approved protocol. Once the medications are discontinued, the side effects tend to clear up naturally in a few weeks. Some, including the author EL, use the sonic hedgehog inhibitors as adjuvant therapy, reducing the size of the basal cell skin cancer, making it more amenable to surgery.

### 3.7 PD‐1 Inhibitors: Cemiplimab (Libtayo)

Cemiplimab is an immunotherapy (PD‐1 inhibitor) approved for advanced BCC that has progressed after hedgehog pathway inhibitors or for patients who are not candidates for surgery. Cemiplimab is administered every 3 weeks via IV (Intravenous) infusion, and side effects are often immune‐related, collectively referred to as “itis” syndromes (pneumonitis, colitis, and dermatitis) [12].

## 4 Topical Therapies

### 4.1 Imiquimod (Aldara)

Imiquimod is an immune response modifier applied topically for superficial BCC. Its success rate is 80% [11]. Although protocols vary, patients typically apply it daily for a total of 6 weeks with occasional breaks taken if a strong inflammatory skin response ensues. The clinical response typically begins after 2 weeks, with redness, oozing, and crusting as common side effects.

### 4.2 5‐Fluorouracil (5‐FU)

5‐FU is also FDA‐approved for superficial BCC. Applied twice daily for 3–6 weeks, it works by inhibiting DNA synthesis in cancerous cells. Like imiquimod, 5‐FU has an 80% success rate but may cause more irritation and crusting at the application site [11].

#### 4.2.1 Comparative Research

Studies have shown that imiquimod may have a slightly better cosmetic outcome than 5‐FU, but patient compliance and side effects vary depending on individual tolerance [9].

## 5 Alternatives (Non‐FDA Approved)

### 5.1 Lasers Targeting Angiogenesis

Both PDL (Pulse Dye Laser) 595 nm wavelength (75% clearance) [13] and Nd:YAG 1064 nm wavelength (90% clearance) [14] lasers, alone and in combination, have shown promise as alternative treatments for select BCCs, particularly non‐aggressive subtypes that were not candidates for Mohs surgery. The mechanism is believed to be the targeting of the blood vessels supplying the BCC and often visible on the surface of the skin cancer. However, further research is still needed to optimize protocols and determine ideal patient selection. Although the studies revealed clinically all tumors shrunk in size, recurrences were rare and related to more aggressive basal cell cancers (infiltrative and morpheaform types) and being located on the face.

### 5.2 Lasers Functioning Through Tissue Vaporization

Pulsed CO_2_ laser resurfacing treatment has been shown to effectively ablate superficial and early nodular BCCs. Treatment of the neoplasm with 2–9 mm margins and 2–3 passes (10,600 nm and fluence of 7.5 J/cm^2^) is recommended for complete vaporization using this laser system [15].

#### 5.2.1 Photodynamic Therapy (PDT)

PDT involves applying aminolevulinic acid (ALA) or methyl aminolevulinate (MAL) to the lesion, followed by exposure to light. This therapy creates reactive oxygen species that destroy cancerous cells. PDT has an 80% success rate for the treatment of superficial BCCs (SBCC). Although it is used in many clinical settings and is approved in Europe, the FDA has not approved it in the U.S. for treating SBCC [1].

#### 5.2.2 Isotretinoin

Although not FDA‐approved for BCC treatment, isotretinoin has shown some efficacy in reducing the size and number of BCCs. However, tumors often recur once the medication is discontinued, so isotretinoin is best used in combination with other therapies [1].

## 6 Prophylaxis and Supplements

There is growing interest in the use of supplements for BCC prevention. A 2015 study published in the New England Journal of Medicine showed a 23% reduction in new BCCs and actinic keratoses (AKs) with niacinamide (500 mg twice daily) [6]. Niacinamide's anti‐inflammatory properties may help reduce UV‐induced immunosuppression, lowering the risk of new skin cancers [6]. Other potential supplements include NMN (Nicotinamide Mononucleotide), a supplement author E.L. uses personally and recommends to all his patients at a 1000 mg per day dose. These supplements may support DNA repair and cellular health, although more research is needed.

## 7 BCC Treatment Algorithm

Since BCCs rarely metastasize, treatment guidelines are based on the tumor's risk of recurrence, patient related factors such as immune status, and ease of achieving clear margins. The European and American guidelines slightly differ in recommendation for surgical margins, follow up protocols, and use of systemic therapies, the latter being less often utilized in Europe. The American guidelines summarized by the National comprehensive cancer network (NCCN) classify treatment based on the risk of local recurrence‐ High Versus Low (see Table 2) suggesting 4 mm excision margins for low risk BCCs and wider margin or mohs for high risk or locally advanced tumors (see Table 2). The European guidelines, on the other hand, categorize BCCs based on ease or difficulty to treat with recommended wider tumor margins of 5–10 mm for high risk tumors and 3–4 mm margins for low risk ones [16].

## Author Contributions

**Sophia Levit:** conceptualization (equal), data curation (equal), formal analysis (equal), resources (lead), writing – review and editing (lead). **Joshua Shoykhet:** data curation (equal), resources (equal), visualization (equal), writing – review and editing (equal). **Eyal Levit:** conceptualization (lead), data curation (lead), formal analysis (lead), funding acquisition (lead), investigation (lead), methodology (lead), project administration (lead), resources (lead), software (lead), supervision (lead), validation (lead), visualization (lead), writing – original draft (lead), writing – review and editing (lead).

## Consent

The authors obtained written consent from patients for their photographs and medical information to be published in print and online and with the understanding that this information may be publicly available. Patient consent forms were not provided to the journal but are retained by the authors. For further ethical considerations or inquiries, please contact Dr. Eyal Levit at eyallevit@gmail.com.

## Conflicts of Interest

The authors declare no conflicts of interest.

## Data Availability

The data that support the findings of this study are available from the corresponding author upon reasonable request.
